# BINGE EATING DISORDER AND THE RISK OF SUICIDE IN OLDER WOMEN

**DOI:** 10.1093/geroni/igae098.2831

**Published:** 2024-12-31

**Authors:** Therese Mendez, Ann O’Hanlon

**Affiliations:** University of New Orleans, New Orleans, Louisiana, United States; University of New Orleans, New Orleans, Louisiana, United States

## Abstract

Binge Eating Disorder (BED) is a psychiatric condition marked by repeated instances of excessive food consumption without control. Research on eating disorders tends to focus on younger populations, but recent studies have shown an increasing prevalence of BED in older women. Binge Eating Disorder may pose unique challenges and heightened risks for older women, including an increased potential for suicidality. This review aims to spotlight the critical intersection of binge eating disorder, its prevalence among older women, and the associated risk of suicide. A scoping literature review was done in accordance with the PRISMA-ScR criteria, targeting studies published between 1996 and 2024 on PubMed, PsychINFO, CINHAL, Web of Science, and Google Scholar. The search focused on research pertaining to women aged 40 and above, exploring the association between BED and suicide risk within this demographic. Of the literature surveyed, there were no studies that focused explicitly on the population of interest. Only eight studies met the inclusion criteria for BED and the risk of suicidality in women. These studies show that BED significantly correlates with an elevated suicidality risk, highlighting a complicated relationship that adversely impacts mental health and possible mortality. Despite acknowledging the general risk BED poses for suicidality, the review underscores a concerning scarcity of research dedicated to understanding BED’s prevalence and specific suicidality risk among older women. The findings reveal a need for further research to delve into the nuanced psychological effects of BED on older women and its contribution to heightened suicide risk.

